# Aesthetic surgery in Ghana: A one-year retrospective case series of procedures and patient profiles in a tertiary hospital^✰^

**DOI:** 10.1016/j.jpra.2026.05.051

**Published:** 2026-06-04

**Authors:** Paa Ekow Hoyte-Williams, Doreen Kwankyewaa Adjei, Pius Agbenorku, Zainab Schumacher, Adae-Aboagye Kwadwo, Paa Kwasi Fiifi-Yankson, Anita Esi Botchway, Bradford W Rockwell, Lionel Dumont

**Affiliations:** a Division of Plastic Surgery, Komfo Anokye Teaching Hospital, School of Medicine and Dentistry, Kwame Nkrumah University of Science and Technology, Kumasi, Ghana; b University of Ghana Medical Center, Accra, Ghana; c Division of Plastic Surgery, University of Utah School of Medicine, Salt Lake City, USA; d Anesthesiology Service, Department of Acute Medicine, Geneva University Hospital, Geneva, Switzerland

**Keywords:** Aesthetic surgery, Global surgery, Sub-saharan africa, Abdominoplasty, Liposuction

## Abstract

**Background:**

Aesthetic surgery is the most frequently performed surgical category worldwide, with increasing demand across Africa. Ghana reflects global trends; however, no published data describe its aesthetic surgery landscape. This study aimed to map aesthetic procedures performed at a large tertiary hospital in Ghana and to characterize the demographic, medical, and social profiles of patients.

**Methods:**

A retrospective case series included patients aged ≥18 years undergoing aesthetic surgery at Komfo Anokye Teaching Hospital, Kumasi, between January 1 and December 31, 2021. Procedures were performed within a tertiary plastic surgery unit involving a limited number of surgeons. Data on demographics, residence, comorbidities, and procedure type were collected. Associations were analyzed using chi-square tests and odds ratios.

**Results:**

Of 156 identified records, 154 met inclusion criteria. One hundred four patients (67.5%) resided in Ghana, while 50 (32.5%) lived abroad, primarily in the United States or Canada, the United Kingdom, and Europe. Mean age was 38.1 years, and 95% of patients were female. Obesity (BMI >30) was present in 66% and was significantly associated with abdominoplasty and Brazilian butt lift (BBL) (p < 0.001), but not liposuction. A total of 385 procedures were performed; 84% of patients underwent multiple procedures. The most common operations were 360° liposuction (82%), BBL (62%), abdominoplasty (44%), and mastopexy (18%). Patients residing outside Ghana underwent abdominoplasty more frequently than local patients (76%vs 28%, p < 0.001). Prior cesarean section was strongly associated with abdominoplasty (72%vs 36%, p < 0.001). Comorbidities were present in 29% of patients, most commonly hypertension and diabetes, without association with procedure type.

**Conclusions:**

This study provides the first overview of aesthetic surgery activity in a tertiary hospital in Ghana. A distinctive procedural profile was observed, dominated by liposuction, BBL, and abdominoplasty, reflecting regional and cultural preferences. These data establish a baseline for future prospective studies evaluating outcomes, complications, training, and safety to inform practice.

## Introduction

Given the central role of body image in modern society, it is unsurprising that individuals seek to modify their appearance to align with prevailing beauty ideals.^1^ Perceptions of beauty, the “ideal” face, and desirable body shape are continuously influenced by media, including advertising, subliminal messaging, and social media platforms.^2^ Globally, aesthetic surgery is gaining popularity for diverse reasons, including reversal of age-related changes.^3^ By definition, aesthetic surgery encompasses surgical and medical procedures aimed at maintaining, restoring, or enhancing physical appearance.^1^

Unlike reconstructive surgery, which addresses morphological changes associated with disease or trauma, aesthetic surgery is primarily life-enhancing rather than lifesaving. Consequently, patients seeking aesthetic procedures often have distinct characteristics, motivations, and expectations compared with those pursuing other medical interventions.^4^ Nonetheless, both aesthetic and reconstructive surgery are integral components of plastic surgery, a discipline that addresses the intertwined goals of physical restoration and psychophysical well-being.^5^^,^^6^

Aesthetic surgery is now the most frequently performed surgical category worldwide, with a prevalence approximately three times higher than reconstructive surgery.^7^ In Africa, demand is rising in parallel with global trends, as demonstrated by both scientific literature^8^, ^9^, ^10^, ^11^ and media reports.^12^^,^^13^ In South Africa, for example, certain surgeons report that up to 80% of their aesthetic procedures are performed on patients from other sub-Saharan countries. The growing availability of aesthetic surgery in major African cities, evidenced by the increasing number of dedicated clinics, suggests that supply is keeping pace with demand.^14^^,^^15^

Advances in medical technology and the rapid expansion of internet-based media have enhanced public awareness and accessibility of aesthetic procedures.^6^^,^^16^ Ghana reflects these global patterns: the number of plastic surgeons and other providers offering aesthetic surgery is steadily increasing, making such procedures more accessible to the population. Despite this rising demand, there are no published data describing the demographics of patients undergoing aesthetic surgery in Ghana, nor the types of procedures most frequently performed. The objective of this study was to retrospectively review aesthetic surgical procedures and patient characteristics at a tertiary hospital in Ghana, providing the first systematic characterization of aesthetic surgery practice in this setting.

## Methods

This retrospective case series reviewed admission records of patients who underwent aesthetic surgery at the Reconstructive Surgical Unit of Komfo Anokye Teaching Hospital (KATH), Kumasi, Ghana, between January 1 and December 31, 2021. KATH is a 1200-bed tertiary care facility and the second largest hospital in the country. Ethical approval was obtained from the KATH Institutional Review Board (KATH IRB/AP/119/22). All patients provided informed consent for the use of their medical data for scientific purposes. All data were anonymized, with no identifiable patient information collected. Electronic files were stored in a password-protected database accessible only to the research team and, if required, the ethics board. Hard-copy documents were kept in locked storage. Given the retrospective design and absence of direct contact with participants, no potential risk to human subjects was anticipated.

All aesthetic surgery cases performed in 2021 were identified and reviewed to extract the relevant data. The study included Ghanaian patients aged 18 years or older who had undergone aesthetic surgery during the study period and whose records contained, at minimum, demographic information and details of the procedures performed. Patients younger than 18 years, non-Ghanaian patients, and those with incomplete essential data were excluded.

Data extracted included demographic characteristics (age, sex, weight, height), type of procedure, presence of comorbidities, and history of substance use. Additional variables included place of residence, professional occupation, and source of referral to KATH for aesthetic surgery. Missing data were noted for each variable.

Thirteen distinct surgical procedures were identified, with some patients undergoing multiple operations during the same anesthetic session. Consequently, the total number of procedures exceeded the number of patients. For analysis, each procedure was recorded separately; for example, a lipoabdominoplasty was counted as both a liposuction and an abdominoplasty (two procedures). The procedures were categorized as follows: breast lift, breast augmentation (with autologous fat or prosthesis), abdominoplasty, 360° liposuction, Brazilian buttock augmentation and lift (BBL), breast reduction, gynecomastia surgery, arm liposuction, thigh liposuction, calf augmentation, scar revision, face/neck/midface lift, and other surgery (hernia repair and keloid injection). The surgical techniques for these procedures have been described in detail elsewhere.

Aesthetic procedures were performed within the Plastic Surgery Unit by a team of consultants and residents. During the study period, the unit comprised five consultants and five residents; however, aesthetic procedures were primarily performed by two consultants. The first author, as head of the unit, was directly or indirectly involved in the majority of cases, either as primary surgeon, co-surgeon, or supervisor.

Descriptive statistics were performed with continuous variables presented as mean (standard deviation) and categorical variables as frequencies with percentages. Missing data were handled using complete case analysis with correlation matrices calculated using pairwise complete observations. Comparisons between patients residing inside versus outside Ghana were performed using Welch's two-sample *t*-test for continuous variables and Pearson's chi-squared test for categorical variables. For contingency tables with expected cell counts <5, Fisher's exact test was used instead of chi-squared test. Statistical significance was set at p < 0.05 for all analyses, and all statistical analyses were performed with R 4.4.3 using the dplyr, ggplot2, and gtsummary packages. The association between abdominoplasty and prior cesarean section was evaluated using chi-square/Fisher’s exact test with odds ratios and 95% CIs.

## Results

A total of 156 patient records were retrieved for analysis. One record from a 12-year-old patient and one incomplete record were excluded, leaving 154 patients for inclusion. Missing data were present for nearly all variables except for type of surgery, which was consistently documented.

Of the study cohort, 104 patients (67.5%) resided in Ghana, while 50 (32.5%) lived abroad, predominantly in the United States/Canada, the United Kingdom, and Europe (table 1). Patient resided inside Ghana were younger (34.8 vs 45.1; p < 0.001) with a lower proportion of obesity (58% vs83%; p < 0.05). Occupational categories included healthcare (25%), business (41%), beauty/fashion (7%), and students (7%), with the remaining 20% classified as other or unknown. Sources of referral to KATH for aesthetic surgery were friends (35%) and the internet (1.3%), while 63% were undocumented.

**Table 1:** Demographic, medical, and surgical characteristics of patients undergoing aesthetic surgery, stratified by residence (inside vs. outside Ghana).

Variable	N	Overall N = 154^1^	Inside Ghana N = 104^1^	Outside Ghana N = 50^1^	P-value^2^
**Age (years)**	154	38.1 (10.2)	34.8 (9.6)	45.1 (7.8)	<0.001*
Min-Max (Median)		18–63 (38)	18–63 (34)	29–56 (45)	
**Weight (kg)**	125	88.3 (13.7)	87.3 (14.8)	90.5(10.9)	0.2
**Sex**	154				>0.9
*Male*		7 (4.5%)	5 (4.8%)	2 (4.0%)	
*Female*		147 (95%)	99 (95%)	48 (96%)	
**BMI Category**	125				0.045*
*Underweight*		1 (0.8%)	1 (1.2%)	0 (0%)	
*Normal*		3 (2.4%)	3 (3.6%)	0 (0%)	
*Overweight*		38 (30%)	31 (37%)	7 (17%)	
*Obese*		83 (66%)	49 (58%)	34 (83%)	
**Medical Comorbidities**	153	44 (29%)	26 (25%)	18 (36%)	0.2
**Surgical Prodecures**	154				
Breast Lift	154	28 (18%)	16 (15%)	12 (24%)	0.3
Breast Augmentation (Fat)	154	5 (3.2%)	3 (2.9%)	2 (4.0%)	>0.9
Breast Augmentation (Prosthesis)	154	0 (0%)	0 (0%)	0 (0%)	
Abdominoplasty	154	67 (44%)	29 (28%)	38 (76%)	<0.001*
360° Liposuccion	154	127 (82%)	83 (80%)	44 (88%)	0.3
Brazilian Butt Lift (BBL)	154	96 (62%)	70 (67%)	26 (52%)	0.1
Breast Reduction	154	4 (2.6%)	3 (2.9%)	1 (2.0%)	>0.9
Gynaecomastia	154	4 (2.6%)	3 (2.9%)	1 (2.0%)	>0.9
Arm Liposuction	154	30 (19%)	26 (25%)	4 (8.0%)	0.023*
Thigh Liposuction	154	10 (6.5%)	7 (6.7%)	3 (6.0%)	>0.9
Calf Augmentation	154	1 (0.6%)	1 (1.0%)	0 (0%)	>0.9
Scar Revision	154	1 (0.6%)	0 (0%)	1 (2.0%)	0.7
Face/Neck Lift	154	3 (1.9%)	0 (0%)	3 (6.0%)	0.057
Other Surgery	154	8 (5.2%)	7 (6.7%)	1 (2.0%)	0.4
Total Surgeries per Patient	154				0.2
*1*		25 (16%)	18 (17%)	7 (14%)	
*2*		52 (34%)	39 (38%)	13 (26%)	
*3*		59 (38%)	38 (37%)	21 (42%)	
*4*		12 (7.8%)	7 (6.7%)	5 (10%)	
*5*		6 (3.9%)	2 (1.9%)	4 (8.0%)	

1 Mean (SD) for continuous variables; n (%) for categorical variables.

2 Pearson’s Chi-squared test; Welch Two Sample *t*-test. N refers to the number of patients with available data for each variable; remaining cases represent missing data.

Overall, 83 patients (66%) were obese (BMI > 30) (Table 1). Fifty-two patients (33.8%) had no prior surgical history, whereas 102 (66.2%) reported previous surgery, including cesarean section (n = 43; 27.7%), bariatric surgery (n = 4; 2.6%), and prior aesthetic surgery (n = 41; 26.5%). Forty-four patients (29%) had significant comorbidities, most frequently hypertension (10%), diabetes mellitus (7.7%), gastrointestinal symptoms (6.5%), anemia, thyroid disease, G6PD deficiency, and neurologic disorders (< 2% each). Regular cigarette and marijuana use were reported in 5.2% and 9.2% of patients, respectively. Alcohol consumption was reported in 52% (occasional: 49%; frequent: 3.2%).

In total, 385 procedures were performed among 154 patients. Only 16% underwent a single procedure, whereas 34% had two, 38.7% had three, 7% had four, and 3.9% had five procedures. The most common operations were 360° liposuction, Brazilian butt lift (BBL), abdominoplasty, and mastopexy. Frequent combinations included liposuction plus BBL (n = 28) and liposuction plus BBL plus abdominoplasty (n = 37). The male patients who sought Gynaecomastia surgery were 5 (1.17%). Those who sought Breast reduction procedure were 4 in number (0.94%). Three patients (0.70%) had Chin lift procedures. One patient (0.23%) sought Calf augmentation, and another (0.23%) sought Scar revision. Four patients (0.94%) had other surgical procedures (hernia repair) in addition to the aesthetic procedures performed

Regression analysis and a correlation matrix were performed to evaluate the influence of all recorded variables on surgical procedure selection. Based on these findings, subgroup analyses were conducted focusing on residence, BMI, and comorbidities.

Residence was significantly associated with both abdominoplasty and arm liposuction. Patients residing outside Ghana underwent abdominoplasty at a markedly higher rate than local patients (p < 0.001) and arm liposuction less frequently (p < 0.05). No other procedure frequencies differed significantly by residence (Table 1). In addition, patients from outside Ghana were significantly older (p < 0.001) and had a higher prevalence of obesity (p = 0.045) compared with those residing within the country.

BMI > 30 was significantly associated with higher rates of abdominoplasty (p = 0.016) and BBL (p = 0.036) compared with non-obese patients (Table 2). Obese patients were also significantly older (p = 0.006) and more likely to reside outside Ghana (p = 0.01) than their non-obese counterparts. BMI did not influence the number of procedures performed per patient.

**Table 2:** Demographic and procedural characteristics of patients undergoing aesthetic surgery, stratified by BMI category (non-obese vs. obese).

Variable	N	Overall N = 125^1^	Non-Obese (BMI≤30), N = 42	Obese (BMI>30), N = 83	P-value^2^
**Residence**	125				0.011*
*Inside Ghana*		84 (67%)	35 (83%)	49 (59%)	
*Outside Ghana*		41 (33%)	7 (17%)	34 (41%)	
**Age (years)**	125	38.0 (10.1)	34.5 (9.3)	39.7 (10.1)	0.006*
**Gender**	125				0.9
*Male*		5 (4.0%)	1 (2.4%)	4 (4.8%)	
*Female*		120 (96%)	41 (98%)	79 (95%)	
**BMI Category**	125				<0.001
**Medical Comorbidities**	125	38 (30%)	9 (21%)	29 (35%)	0.2
**Surgical Prodecures**	125				
Breast Lift	125	23 (18%)	7 (17%)	16 (19%)	>0.9
Breast Augmentation (Fat)	125	4 (3.2%)	2 (4.8%)	2 (2.4%)	0.9
Breast Augmentation (Prosthesis)	125	0 (0%)	0 (0%)	0 (0%)	
Abdominoplasty	125	56 (45%)	12 (29%)	44 (53%)	0.016*
360° Liposuccion	125	106 (85%)	36 (86%)	70 (84%)	>0.9
Brazilian Butt Lift (BBL)	125	81 (65%)	33 (79%)	48 (58%)	0.036*
Breast Reduction	125	3 (2.4%)	1 (2.4%)	2 (2.4%)	>0.9
Gynaecomastia	125	2 (1.6%)	1 (2.4%)	1 (1.2%)	>0.9
Arm Liposuction	125	23 (18%)	8 (19%)	15 (18%)	>0.9
Thigh Liposuction	125	9 (7.2%)	4 (9.5%)	5 (6.0%)	0.7
Calf Augmentation	125	1 (0.8%)	0 (0%)	1 (1.2%)	>0.9
Scar Revision	125	1 (0.8%)	0 (0%)	1 (1.2%)	>0.9
Face/Neck Lift	125	3 (2.4%)	1 (2.4%)	2 (2.4%)	>0.9
Other Surgery	125	5 (4.0%)	3 (7.1%)	2 (2.4%)	0.4

1 Mean (SD) for continuous variables; n (%) for categorical variables.

2 Pearson’s Chi-squared test; Welch Two Sample *t*-test. N refers to the number of patients with available data for each variable; remaining cases represent missing data.

Comorbidities had no significant impact on the type of procedure performed (Table 3). However, patients with comorbidities were significantly older than those without (p = 0.004). No associations were found between procedure type and history of substance use (tobacco, alcohol, marijuana), previous surgery in general, or previous aesthetic surgery specifically. In contrast, a strong association was observed between prior cesarean section and abdominoplasty: 72% (31 of 43) of patients with a cesarean section history underwent abdominoplasty compared with 36% (36 of 112) without such history (OR, 5.38; 95% CI, 2.48–11.69; p < 0.001).

**Table 3:** Demographic and procedural characteristics of patients undergoing aesthetic surgery, stratified by medical comorbidities (No comorbidities vs. Has comorbidities).

Variable	N	Overall N = 154^1^	No comorbities, N = 110	Has comorbities, N = 44^1^	P-value^2^
**Residence**	154				0.2
***Inside Ghana***		104 (67%)	77 (71%)	27 (59%)	
***Outside Ghana***		50 (33%)	32 (29%)	18 (41%)	
**Age (years)**	154	38.2 (10.2)	36.7 (10.0)	41.9 (9.9)	0.004*
**Gender**	154				0.7
***Male***		7 (4.5%)	4 (3.6%)	3 (6.8%)	
***Female***		147 (95%)	106 (96%)	41 (93%)	
**Obese status**	125				0.2
***Non-Obese (BMI≤30)***		42 (34%)	33 (38%)	9 (24%)	
***Obese (BMI>30)***		83 (66%)	54 (62%)	29 (76%)	
**Surgical Procedures**					
**Breast Lift**	154	28 (18%)	17 (15%)	11 (25%)	0.2
**Breast Augmentation (Fat)**	154	5 (3.2%)	3 (2.7%)	2 (4.5%)	>0.9
**Breast Augmentation (Prosthesis)**	154	0 (0%)	0 (0%)	0 (0%)	
**Abdominoplasty**	154	67 (44%)	45 (41%)	22 (50%)	0.4
**360° Liposculpture**	154	127 (82%)	92 (84%)	35 (80%)	0.7
**Brazilian Butt Lift (BBL)**	154	97 (63%)	75 (68%)	22 (50%)	0.054
**Breast Reduction**	154	4 (2.6%)	2 (1.8%)	2 (4.5%)	0.7
**Gynaecomastia**	154	4 (2.6%)	2 (1.8%)	2 (4.5%)	0.7
**Arm Liposuction**	154	30 (19%)	21 (19%)	9 (20%)	>0.9
**Thigh Liposuction**	154	10 (6.5%)	8 (7.3%)	2 (4.5%)	0.8
**Calf Augmentation**	154	1 (0.6%)	0 (0%)	1 (2.3%)	0.6
**Scar Revision**	154	1 (0.6%)	1 (0.9%)	0 (0%)	>0.9
**Face/Neck Lift**	154	3 (1.9%)	1 (0.9%)	2 (4.5%)	0.4
**Other Surgery**	154	8 (5.2%)	7 (6.4%)	1 (2.3%)	0.5

1 Mean (SD) for continuous variables; n (%) for categorical variables.

2 Pearson’s Chi-squared test; Welch Two Sample *t*-test. N refers to the number of patients with available data for each variable; remaining cases represent missing data.

**Fig. 1:**
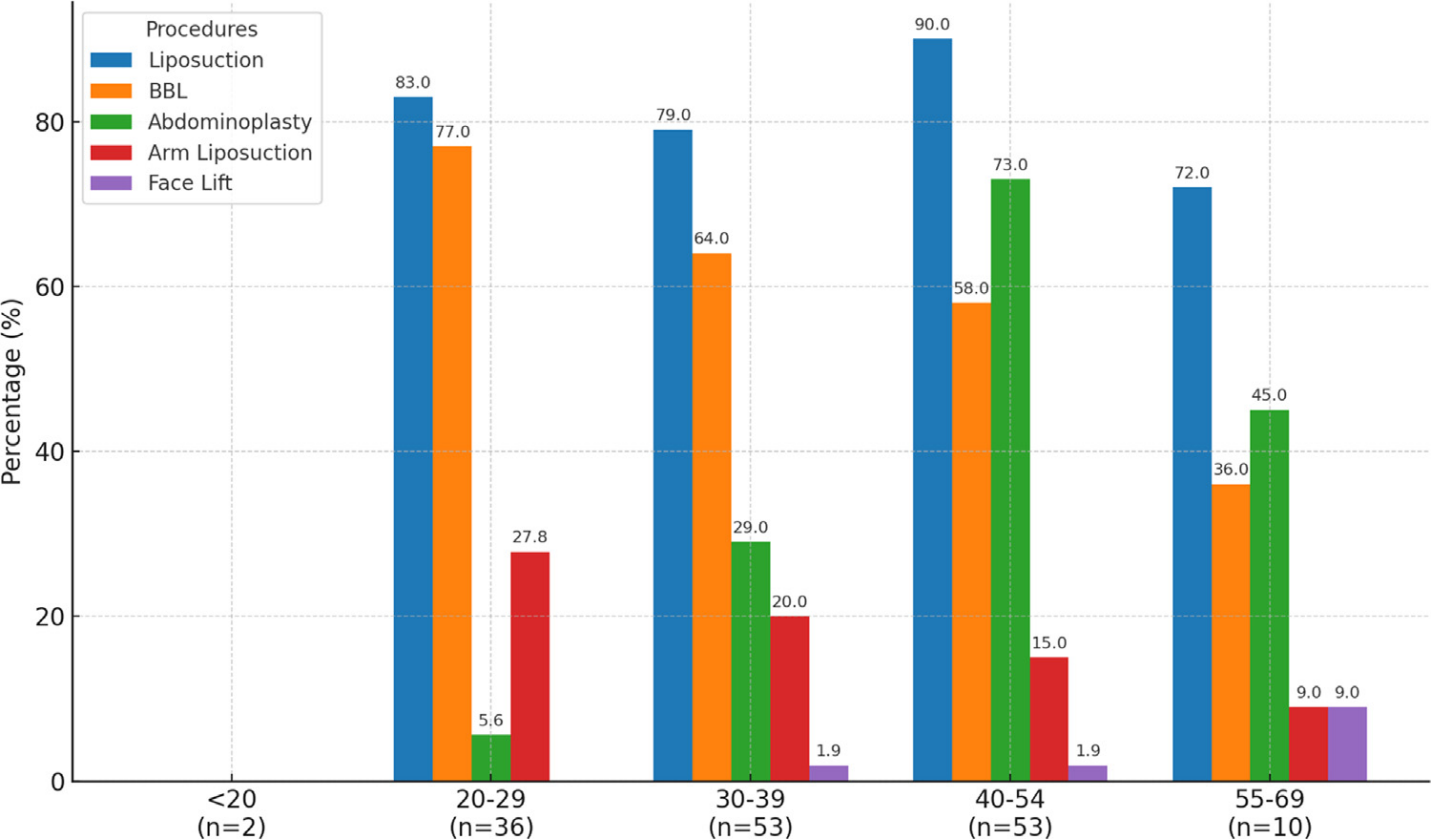
Main Procedure Patterns by Age Group. Main procedure patterns by age group. Percentages represent the proportion of patients within each age category undergoing the listed procedure (n = number of patients per age group). Because patients could undergo multiple procedures during the same anesthetic session, percentages within groups may exceed 100%.

Analysis of procedure distribution by age group (n = 154) showed distinct patterns (Fig. 1). Two patients (1.3%) were younger than 20 years; one underwent gynecomastia surgery and the other macrodactyly correction, both classified as “other procedures.” Among patients aged 20–29 years (n = 36; 23.4%), liposuction was most frequent (83%), followed by BBL (77%), arm liposuction (27.8%), and abdominoplasty (5.6%); no facial procedures were performed in this group. In the 30–39-year group (n = 53; 34.4%), liposuction (79%) and BBL (64%) remained predominant, with abdominoplasty performed in 29% and arm liposuction in 20%. In patients aged 40–54 years (n = 53; 34.4%), liposuction was performed in 90%, abdominoplasty in 73%, and BBL in 58%, with arm liposuction in 15% and face lift in 1.9%. Among the oldest group, aged 55–69 years (n = 10; 6.5%), liposuction was performed in 72%, abdominoplasty in 45%, BBL in 36%, arm liposuction in 9%, and face lift in 9%. Across all age groups, liposuction remained the most common procedure, with abdominoplasty and BBL consistently ranking in the top three.

## Discussion

This study provides, for the first time, a comprehensive mapping of aesthetic surgery practice at a major tertiary hospital in Ghana, encompassing procedure types, as well as demographic, medical, and social characteristics of the patient population.

From a procedural perspective, our findings partially mirror trends observed in high-income countries, where liposuction and abdominoplasty consistently rank among the most frequently performed aesthetic procedures.^17^ However, the distribution differs markedly from Western norms. Breast augmentation represented <3% of all cases, and no blepharoplasty procedures were recorded. These discrepancies highlight the influence of cultural and regional factors in shaping aesthetic preferences.^18^ This is further illustrated by the high prevalence of Brazilian butt lift (BBL) procedures, performed in 62% of patients, despite this operation not ranking among the top 10 aesthetic procedures in the United States.^17^

The majority of patients were aged 30–39 or 40–49 years, together comprising over 60% of the cohort. Direct comparison with international trends is challenging due to temporal shifts in procedure popularity^19^^,^^20^ and significant variation by procedure type.^21^ Age-specific analysis revealed that in patients aged 20–29 years, liposuction and BBL predominated, in contrast to Western data where breast augmentation and facial surgery are more common. In the 30–39 year group, abdominoplasty joined liposuction and BBL as the leading procedures, whereas in the West, this age group more closely resembles the 20–29 year cohort.^17^ Notably, even in patients aged 55–69 years (an age group in Western practice largely focused on facial rejuvenation) the abdominoplasty–BBL–liposuction triad remained dominant.^17^ These findings differ from trends in Asia and the Middle East, where facial procedures such as rhinoplasty, blepharoplasty, and nonsurgical injections are prevalent,^22^^,^^23^ and are more closely aligned with South American patterns, particularly in Colombia, where liposuction and BBL are also highly represented.^24^

As reported globally, the overwhelming majority of patients were female (95%).^3^^,^^17^ Most were obese (BMI > 30), and obesity was significantly associated with higher rates of abdominoplasty and BBL, though not with liposuction. Published data on aesthetic surgery in obese, non-bariatric populations remain scarce.^25^^,^^26^ For abdominoplasty in particular, complication rates are known to be higher in obese, non-bariatric patients.^27^

Medical comorbidities were not correlated with the type of procedure, yet 29% of patients presented with at least one comorbidity—unsurprising given the high prevalence of obesity and the fact that 40% of patients were older than 40 years. This raises important considerations regarding elective, non-medically indicated surgery in a population with substantial rates of hypertension, diabetes, and other risk factors, all of which may increase the likelihood of medical complications.^28^

Interestingly, patient residence emerged as a strong differentiator in demographic and procedural profiles. Ghanaian residents were younger, more often obese, underwent fewer abdominoplasties, and had a higher proportion of arm liposuction. All facial lifting procedures were performed in non-residents. The growing field of aesthetic surgery tourism likely contributes to this pattern, with Ghanaian surgeons attracting patients from abroad—particularly from Europe and North America—where costs for comparable procedures are substantially higher.^29^^,^^30^

Occupational data suggest that this patient population largely represents a high socioeconomic stratum, consistent with global trends.^31^^,^^32^ While the rise of internet and social media is widely cited as a driver of global demand for aesthetic surgery,^2^ in our cohort, friend referrals were by far the most common source of surgeon selection, with internet sources accounting for only 1.3% of referrals.

A particularly noteworthy finding was the strong association between prior cesarean delivery and abdominoplasty. Women with a cesarean history were more than five times more likely to undergo abdominoplasty, consistent with prior reports linking cesarean delivery to both aesthetic motivations and increased postoperative risk.^33^

This study has several limitations. First, this was a single-center retrospective study in which aesthetic procedures were performed by a limited number of surgeons within one tertiary unit. Although several consultants were part of the team, only two were regularly involved in aesthetic procedures, and the first author, as head of the unit, was directly or indirectly involved in most cases. As such, the distribution of procedures may partly reflect surgeon-specific expertise, preferences, and scope of practice. However, this should be interpreted within the national context, where plastic and aesthetic surgery services were extremely limited during the study period, with only a few providers concentrated mainly in Accra.^34^ Our institution served as the primary referral center for the middle and northern regions of Ghana, representing a large proportion of the accessible national practice at that time.

In addition, the retrospective, single-center design relied on incomplete records, with missing data for key variables such as height, weight, and BMI. Complications and long-term outcomes were not systematically recorded, limiting meaningful comparison with international safety standards.^35^ Patient-reported outcomes were not captured, limiting assessment of satisfaction and quality of life. Moreover, the one-year study period prevents evaluation of temporal trends. These limitations highlight the need for prospective, multicenter studies with standardized reporting of complications, outcomes, and qualitative patient perspectives.

In conclusion, this study provides the first systematic mapping of aesthetic surgery practice in a tertiary hospital in Ghana. It documents a distinctive pattern of aesthetic procedure shaped by cultural, demographic, and regional factors, and identifies key priorities for future research, notably the roles of obesity and prior cesarean delivery, as well as the need for standardized reporting of outcomes and complications in sub-Saharan Africa.

## Funding

This study received no external funding.

## Ethical approval

Ethical approval was obtained from the KATH Institutional Review Board of the Komfo Anokye Teaching Hospital (KATH) (KATH IRB/AP/119/22).

## Declaration of generative AI and AI-assisted technologies in the manuscript preparation process

During the preparation of this manuscript, the authors used ChatGPT 5.1 (OpenAI) to assist with language editing and to improve the clarity and quality of written English for non-native English speakers. After using this tool, the authors carefully reviewed and edited the content as needed and take full responsibility for the accuracy, integrity, and originality of the published article.

## Conflict of Interest

The authors declare no conflicts of interest.
